# Analysis of the Negative-SET Behaviors in Cu/ZrO_2_/Pt Devices

**DOI:** 10.1186/s11671-016-1762-5

**Published:** 2016-12-07

**Authors:** Sen Liu, Xiaolong Zhao, Qingjiang Li, Nan Li, Wei Wang, Qi Liu, Hui Xu

**Affiliations:** 1 College of Electronic Science and Engineering, National University of Defense Technology, Changsha, 410073 China; 2 Key Laboratory of Microelectronic Devices and Integrated Technology, Institute of Microelectronics, Chinese Academy of Sciences, Beijing, 100029 China

**Keywords:** Electrochemical metallization memory, Resistive switching, Conductive filament, Temperature dependence, Oxygen vacancy

## Abstract

Metal oxide-based electrochemical metallization memory (ECM) shows promising performance for next generation non-volatile memory. The negative-SET behavior has been observed in various oxide-based ECM devices. But the underlying mechanism of this behavior remains unaddressed and the role of the metal cation and oxygen vacancy in this behavior is unclear. In this work, we have observed two kinds of negative-SET (labeled as N-SET1 and N-SET2) behaviors in our Cu/ZrO_2_/Pt devices. Both the two behaviors can result in hard breakdown due to the high compliance current in reset process. The *I-V* characteristic shows that the two negative-SET behaviors have an obvious difference in operation voltage. Using four-probe resistance measurement method, the resistance-temperature characteristics of the ON-state after various negative-SET behaviors have been studied. The temperature dependence results demonstrate that the N-SET1 behavior is dominated by Cu conductive filament (CF) reformation caused by the Cu CF overgrowth phenomenon while the N-SET2 is related to the formation of oxygen vacancy CF. This work may provide a comprehensive understanding of the switching mechanism in oxide-based ECM devices.

## Background

The resistive switching (RS) phenomenon, occurred in resistive random access memory (RRAM) also named memristive device, has been widely studied for various applications [1–7]. The binary metal oxide RRAM, which consists of an insulating layer sandwiched between two metal electrodes, has been considered one of the most promising candidate for the next-generation non-volatile memory due to the simple structure and high performance [8–10]. According to the electrochemical activeness of the electrode and switching mechanism, the metal oxide RRAM is divided into electrochemical metallization memory (ECM), valence change memory (VCM), and thermochemical memory (TCM) [11, 12]. The ECM device, also called conductive bridge random access memory (CBRAM), consists of an electrochemical active electrode (e.g., Cu, Ag, or Ni) and an inert electrode (e.g., Pt, W, or Au). The resistance switching phenomenon of ECM device is dominated by the formation and dissolution of conductive filament (CF) which is relative to the redox process of the active metal atoms [13–15]. The VCM device consists of two metal electrodes which are not easily oxidized or the oxidized form are not easily reduced back to the metal atom. The resistive switching of VCM device is triggered by a drift of oxygen ion-related defects, typically oxygen vacancies [11]. While TCM effect is used to explain the unipolar phenomenon in the metal oxide RRAM, in which a current-induced temperature increase leads to a fuse-antifuse process.

According to the ECM theory, a CF consisting of active electrode atoms can be formed in the ECM device when a positive voltage is applied on the active electrode in forming (P-Forming) or set (P-SET) process. Vice versa, the CF will be dissolved when a negative voltage is applied on the active electrode in reset (N-RESET) process. As a result, the ECM device will be operated in bipolar switching mode. In addition, when a negative voltage is applied to a virgin oxide-based ECM device, which is called negative forming (N-Forming) process, an oxygen vacancy CF can also be formed to bridge the electrodes [16, 17]. The ECM effect and VCM effect are coexistent in the oxide-based ECM device due to the inevitable oxygen vacancies in the oxide layer, especially when a negative voltage is applied on the device [18, 19]. Recently an unexpected negative-SET (N-SET) behavior has been observed in several ECM structure devices [20–24], which means that a CF is formed in the negative voltage. The N-SET behavior will cause an overset issue under DC sweeping mode due to the high compliance current in N-RESET process, resulting in hard breakdown. However, the underlying mechanism of the N-SET behavior remains unaddressed and the role of the metal cation and oxygen vacancy in the CF formation process at the negative voltage is unclear.

In our previous work, we have demonstrated that the metallic CF overgrowth in ECM devices can cause N-SET behavior [24]. While in this work, we have further observed two kinds of N-SET behaviors (labeled as N-SET1 and N-SET2) in our Cu/ZrO_2_/Pt devices. The operation voltages of the N-SET1 and N-SET2 behaviors are ~−2.5 and ~−4 V, respectively. Both the N-SET1 and N-SET2 behaviors can result in hard breakdown. The statistical results of operation voltages extracted from 80 devices show that the voltage distribution of N-RESET and N-SET1 have an obvious overlap and the voltage distribution of N-SET2 is close to that of N-Forming. In order to reveal the nature of the CFs, the temperature dependences of the CFs after N-SET1 and N-SET2 behaviors were measured to compare with that of the P-Forming and N-Forming devices. The results show that the CF of N-SET1 behavior has a similar temperature coefficient with that of P-Forming behavior, while the CF of N-SET2 behavior has a close temperature coefficient with that of N-Forming behavior. Based on these results, we demonstrated that the N-SET1 behavior is dominated by Cu CF reformation caused by the Cu CF overgrowth phenomenon while the N-SET2 is related to the formation of oxygen vacancy CF. Finally, a mechanism based on the coexistence of the ECM and VCM effects is proposed to explain the two kinds of N-SET behaviors.

## Methods

**Fig. 1:**
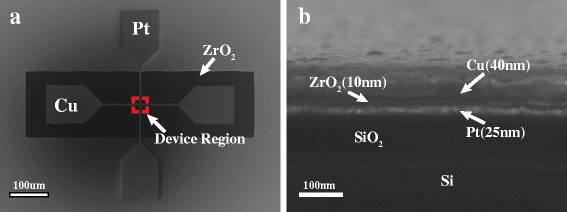
SEM images of the as-fabricated devices. **a** SEM image of the device with crossbar structure. Device area is 25 μm^2^ (5 × 5 μm). The ZrO_2_ layer is patterned to uncover the Pt bottom electrodes. **b** Cross-sectional SEM image of the Cu/ZrO_2_/Pt device. The thickness of ZrO_2_ layer is 10 nm

The ECM devices with the structure Cu/ZrO_2_/Pt were fabricated as follows. First, the vertical lines of Pt/Ti (25/5 nm) were deposited on the SiO_2_/Si substrate by e-beam evaporation after the first lithography process. A thin Ti layer (5 nm) was used to enhance the adhesion of Pt and SiO_2_ layers. Then, the insulting oxide layer of ZrO_2_ (10 nm) was grown by magnetron sputtering after the second lithography process. The ZrO_2_ layer was patterned to uncover the Pt pad, which facilitates to minimize the sheet resistance of the probe and Pt pad. After the third lithography process, horizontal lines of the Pt/Cu (10/40 nm) were deposited by magnetron sputtering. The Pt layer (10 nm) was covered on Cu layer to prevent the oxidation of Cu. The cell areas of the devices range from 4 μm^2^ (2 × 2 μm) to 25 μm^2^ (5 × 5 μm). The scanning electron microscope (SEM) image of the as-fabricated device and the cross-section SEM image of the device region are shown in Fig. 1.

The *I-V* characteristics of the devices were measured with Agilent B1500 semiconductor parameter analyzer. During the electrical measurement, the voltage was applied to the Cu electrode while the Pt electrode was tied to ground. The 1- and 10-mA compliance currents were applied in set and reset process to achieve reproducible switching cycles respectively. The four-probe resistance measurement was applied to obtain the accurate ON-state resistance of the device by eliminating the effect of the electrode line resistance and sheet resistance of the probe and pad. The temperature dependence of the ON-state of the device was performed in the temperature of 300 to 410 K.

## Results and Discussion

**Fig. 2:**
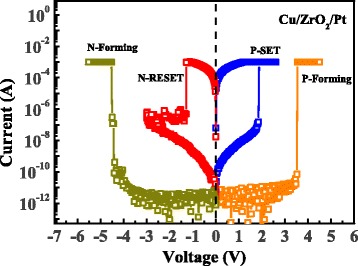
Typical *I-V* curves of the resistive switching in the Cu/ZrO_2_/Pt devices. The 1-mA compliance current is used in P-Forming, P-SET, and N-Forming processes to achieve stable switching cycles. The 10-mA compliance current is used in N-RESET process

The typical *I-V* curves of the Cu/ZrO_2_/Pt devices are shown in Fig. 2. The initial resistance of the virgin device is ~10^11^ Ω at 0.1-V read voltage. Similar to other ECM devices, a forming process is required to achieve reproducible switching cycles [25, 26]. When a positive voltage sweeping (0 → 4.5 V) was applied on a virgin device, a current abrupt can be observed at about 3.3 V and then the device switched to ON-state. After P-Forming process, the Cu/ZrO_2_/Pt device shows bipolar resistive switching behavior and the device can switch between ON-state and OFF-state repeatedly by N-RESET (0 → −3 V) and P-SET (0 → 2.5 V) process, as shown by the blue and red lines in Fig. 2. The typical resistances of the ON-state and OFF-state are on the order of 10^2^ and 10^10^ Ω, respectively. It is worth noting that the device can also switch to ON-state at ~−4.5 V when a negative voltage sweep (0 → −5.5 V) was applied on a virgin device in the N-Forming process, as shown by the dark yellow line in Fig. 2.

**Fig. 3:**
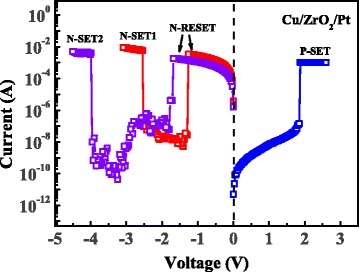
N-SET behaviors of the Cu/ZrO_2_/Pt devices in the N-RESET process under DC sweep mode. Two kinds of N-SET behaviors under DC voltage sweep mode can be observed. The devices can hardly switch back to OFF-state after N-SET1 or N-SET2 behavior due to the 10-mA compliance current in reset sweeping process

**Fig. 4:**
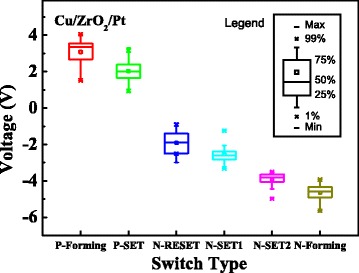
Statistical charts of the operation voltages. These results are statistical data extracted from 80 devices. *Unfilled square* indicates the mean value of the distribution. The voltage distribution of N-SET1 has an obvious overlap with that of N-RESET and the distribution of N-SET2 is close to N-Forming

During the switching cycles, two kinds of N-SET (N-SET1 and N-SET2) behaviors were occasionally observed after N-RESET behavior, as shown in Fig. 3. The N-SET1 behavior occurred at ~−2.5 V, while the N-SET2 occurred at ~−4 V after N-RESET behavior. The device can hardly switch back to OFF-state after N-SET1 or N-SET2 behavior due to the high compliance current in the reset sweeping process. Then the statistical characteristics of the various operation voltages extracted from 80 Cu/ZrO_2_/Pt devices are shown in Fig. 4. Specifically, the device is triggered by the P-Forming (0 → 4.5 V) process. Then, 15 switching cycles with N-RESET (0 → −3 V) and P-SET (0 → 2.5 V) processes are implemented on each device until the N-SET1 behavior happens. If the N-SET1 behavior has not been observed after the 15 switching cycles, another N-RESET process (0 → −5.5 V) is applied on the device following with P-SET process (0 → 2.5 V). The operation voltages of P-Forming, P-SET, N-RESET, N-SET1, and N-SET2 are extracted from the 65 Cu/ZrO_2_/Pt devices. In addition, the operation voltage of N-Forming is extracted from the other 15 Cu/ZrO_2_/Pt devices with N-Forming process (0 → −5.5 V). As a result, the N-SET1 behaviors have been observed in 24 of the 65 devices after 15 switching cycles. But it should be noted that the occurrence probability of N-SET1 increases with the switching cycles. The statistical results demonstrate that the voltage distributions of N-RESET and N-SET1 have an obvious overlap, indicating that the N-SET1 behavior cannot be avoided by controlling the sweeping voltage in N-RESET process. In addition, the results reveal that the voltage distributions of N-SET1 and N-SET2 have no overlap and the mean value of the N-SET1 and N-SET2 are ~−2.6 and ~−4 V, respectively. The obvious voltage difference of the N-SET1 and N-SET2 behaviors indicate that the underlying mechanisms of the two behaviors may be different.

**Fig. 5:**
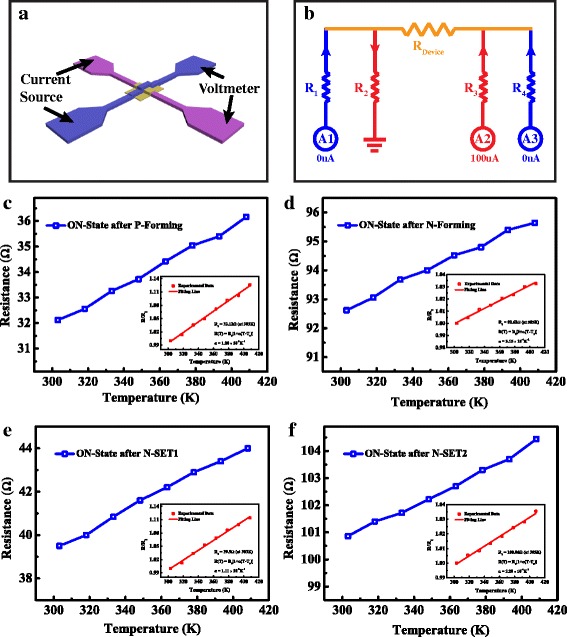
Temperature dependence of the ON-state resistance in Cu/ZrO_2_/Pt devices. **a** Schematic illustration of the test scheme with four-probe resistance measurement method. **b** Equivalent circuit of the crossbar structure and the configuration of the source measurement units (SMUs) in semiconductor parameter analyzer (Agilent B1500) with four-probe resistance measurement method. *R*_Device_ is the ON-state resistance of the device region. The *R*_1_, *R*_2_, *R*_3_, and *R*_4_ are the equivalent resistances (line resistance and contact resistance) of the four pads, respectively. **c**–**f** Typical resistance-temperature characteristics of the ON-state resistances after P-Forming, N-Forming, N-SET1, and N-SET2 behaviors, respectively. The *insets* show the temperature coefficients of the four behaviors at a reference temperature *T*_0_ = 303 K, respectively

To further clarify the underlying mechanism, the resistance-temperature characteristics of the ON-state after various operation behaviors have been studied. The four-probe resistance measurement is used to eliminate the effect of the line resistance and contact resistance [27], and the schematic illustration is shown in Fig. 5a. The equivalent circuit of the crossbar structure is shown in Fig. 5b, in which *R*_Device_ is the device resistance and *R*_1_, *R*_2_, *R*_3_, and *R*_4_ are the total resistances of line resistances and contact resistances of the four electrode pads, respectively. Four source measurement units (SMUs) are used to implement with four-probe resistance measurement and the configurations of the SMUs are shown in Fig. 5b. The use of four-probe resistance measurement method makes it possible to measure the accurate resistance of the device along with the change of temperature. The ON-state resistances after P-Forming, N-Forming, N-SET1, and N-SET2 behaviors have been measured as a function of temperature ranging from 300 to 410 K, as shown in Fig. 5c–f. All the ON-state resistances show a linear increase with temperature, which is typical for electronic transport in metals. The metallic resistance as a function of temperature change can be written as $\mathrm{R}\left(\mathrm{T}\right)={R}_0\left[1+\alpha \left(T-{T}_0\right)\right]$ [28, 29], in which *R*_0_ is the resistance at temperature *T*_0_ and *α* is the resistance-temperature coefficient. Thus, the typical temperature coefficients of the ON-state resistances after P-Forming, N-Forming, N-SET1, and N-SET2 behaviors can be calculated to 1.2 × 10^−3^ K^−1^, 3.15 × 10^−4^ K^−1^, 1.11 × 10^−3^ K^−1^, and 3.29 × 10^−4^ K^−1^ at a reference temperature *T*_0_ = 303 K, respectively. As a result, the ON-state devices after P-Forming and N-SET1 behaviors have similar temperature coefficients which are consistent with the characteristics of Cu CF in the previous literature [30, 31]. While the temperature coefficients of the ON-state devices after N-Forming and N-SET2 behaviors, which are obviously smaller than that of P-Forming and N-SET1, are consistent with the characteristics of oxygen vacancy CF in the previous works [32, 33]. The results indicate that the main ingredients of the CFs after N-SET1 and N-SET2 behaviors are Cu atoms and oxygen vacancy, respectively.

**Fig. 6:**
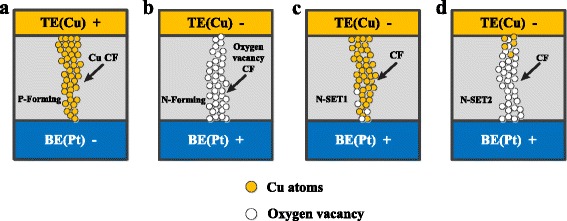
Schematic illustration of the underlying working mechanism of the Cu/ZrO_2_/Pt device. **a** The CF illustration after P-Forming process. A Cu CF is formed to bridge the two electrodes. **b** The CF illustration after N-Forming process. An oxygen vacancy CF is formed to bridge the two electrodes. **c** The CF illustration after N-SET1 behavior. The main ingredient of the CF is Cu atoms. **d** The CF illustration after N-SET2 behavior. The main ingredient of the CF is oxygen vacancy

Here, we propose a microscopic model to describe the underlying switching mechanism in our Cu/ZrO_2_/Pt devices. The virgin Cu/ZrO_2_/Pt device can be formed in both positive and negative voltage polarities. More specifically, when a positive voltage is applied on a virgin device in the P-Forming process, the Cu atoms are oxidized into Cu^2+^ and reduced to Cu atoms inside the ZrO_2_ layer [13, 24]. Then, a Cu CF is formed via electrochemical reaction and electromigration inside the ZrO_2_ layer, as shown in Fig. 6a. Vice versa, when a negative voltage is applied on a virgin device in the N-Forming process, an oxygen vacancy CF will be formed due to the oxygen ion diffusion and migration [16, 17], as shown in Fig 6b. Generally, when the devices are triggered by P-Forming, the ECM and VCM effects are coexistent in the switching cycles. During the N-RESET process, the Cu CF is dissolved due to the thermal-assisted electrochemical reaction by applying a negative voltage on the device [13]. But the N-SET1 behavior may occasionally occur when the Cu atoms precipitated in the inert electrode after several switching cycles provide sufficient Cu^2+^ source to reform the Cu CF in the N-RESET process [24], as shown in Fig. 6c. Otherwise, the N-SET2 behavior can be observed at a higher negative voltage than that of N-SET1. The VCM effects dominate the formation of the oxygen vacancy CFs in the devices, as shown in Fig. 6d. Due to the high compliance current, the device can hardly turn to OFF-state when the two N-SET behaviors occurred.

## Conclusions

In summary, two kinds of N-SET behaviors have been observed in our Cu/ZrO_2_/Pt devices. The detailed *I-V* characteristics reveal that the two N-SET behaviors have an obvious different voltage and the N-SET behaviors may cause hard breakdown because of the high compliance current in the N-RESET process. Subsequently, the statistical results demonstrate that the N-SET1 voltage distribution has an overlap with that of N-RESET, indicating that the N-SET1 behavior cannot be avoided by controlling the operation voltage. The following resistance-temperature characteristics of the four ON-state devices reveal that the main ingredients of the CFs after P-Forming and N-SET1 behaviors are Cu atoms, while the main ingredient of the CFs after N-Forming and N-SET2 behaviors is oxygen vacancy. The results further confirm that the ECM effect and VCM effect are coexistent in our devices. Although the work is implemented with Cu/ZrO_2_/Pt device structure, the experimental method can be easily extended to other ECM devices.

## Abbreviations

CBRAM: Conductive bridge random access memory
CF: Conductive filament
ECM: Electrochemical metallization memory
N-Forming: Negative forming
N-RESET: Negative reset
N-SET: Negative-SET
P-Forming: Positive forming
P-SET: Positive set
RRAM: Resistive random access memory
RS: Resistive switching
SEM: Scanning electron microscope
SMU: Source measurement unit
VCM: Valence change memory
